# A Retrospective Chart Review: The Presence of Anterior Hyaloid Separation Sign in Posterior Vitreous Detachment

**DOI:** 10.7759/cureus.78342

**Published:** 2025-02-01

**Authors:** Peeradol Wattanasirakul, Matthew T Hirabayashi, Raiyan Yousuf, Ahmed Elkeeb

**Affiliations:** 1 Ophthalmology, University of Missouri School of Medicine, Columbia, USA; 2 Ophthalmology, Parkhurst NuVision, San Antonio, USA

**Keywords:** anterior hyaloid separation sign, detection method, diagnosis, posterior vitreous detachment, weiss ring

## Abstract

Background and objective

The presence of Weiss ring is classically used to aid in the diagnosis of posterior vitreous detachment (PVD) but may at times be challenging to visualize under slit-lamp biomicroscopy. The anterior hyaloid separation sign (AHSS) is a previously undescribed sign that is seen among patients with PVD as a "veil of vitreous" in the anterior vitreous cavity. AHSS is easier to visualize than Weiss ring and may prove useful in settings where Weiss ring detection is not feasible.

Methods

This study analyzed the connection between the presence of AHSS among the PVD population. A retrospective chart review of patients with established PVD (n=214) enrolled at Mason Eye Clinic (Columbia, Missouri, United States) between February 1, 2023, and October 1, 2023, was performed. All patients with a confirmed diagnosis of PVD were stratified as exhibiting AHSS or not exhibiting AHSS. Data including patient demographics, lens status, and ocular comorbidities were collected.

Results

The mean age was 70.3 years, 72% were females, and 93.9% were Caucasians. Ocular comorbidities include epiretinal membrane (17.8%), retinal tear (9.8%), proliferative diabetic retinopathy (5.1%), retinal vein occlusion (4.7%), uveitis (4.7%), rhegmatogenous retinal detachment (4.2%), and sickle cell disease (1%). Around 56.2% of cases were newly diagnosed PVD, while 43.8% were long-standing PVD. Among all PVD cases (n=214), 89.7% (n=192) presented with AHSS, whereas 10.3% (n=22) did not present with AHSS. The chi-squared goodness-of-fit test compared this study's AHSS prevalence in PVD with the general PVD population. Analysis revealed a significant prevalence between the presence and absence of AHSS in PVD patients at 84% (p=0.02).

Conclusion

The presence of AHSS has a high prevalence among patients with PVD and holds value in aiding the diagnosis and confirmation of PVD.

## Introduction

Posterior vitreous detachment (PVD) is the separation of the posterior cortical vitreous from the internal limiting membrane of the retina [1]. PVD is most commonly caused by age-related liquefaction and gravity-induced collapse of the vitreous body [2]. The initial separation occurs superiorly and progresses to a rapid separation of the vitreous cortex from the retina, starting in the perifoveal macula [3,4]. Postmortem studies indicate that PVD is present in 27% of eyes by the seventh decade and 63% of eyes by the eighth decade of life [5]. Similarly, clinical studies show that the incidence of PVD is 53% after 50 years and rises to 65% after 65 years of life [6]. Patients with PVD most commonly present with photopsia (flashes of light) and myodesopsia (floaters) with the latter shown to have a negative impact on health-related quality of life [7,8]. Severe complications secondary to PVD can occur due to traction on retinal tissue. Traction involving the retinal vessels can lead to spontaneous hemorrhage within the posterior chamber which may result in rapid clot formation. These clots have slow clearance and can progress to conditions like hemolytic and hemosiderotic glaucoma [9]. Rhegmatogenous retinal detachment (RRD) is another serious complication of PVD. As the cortical vitreous separates from the retinal surface, especially when there is significant adhesion between the vitreous and retina, a full-thickness break may occur which can progress to RRD [10]. RRD is the most common retinal emergency and results in blindness if left untreated [11].

Classically, the presence of Weiss ring seen under ophthalmoscopy is an important sign for the diagnosis of PVD. Weiss ring is defined as peripapillary glial tissue that remains adherent to the posterior vitreous cortex after the posterior vitreous separates from the optic nerve. It can be visualized as an annular vitreous opacity that, when present, is usually found in front of the optic disk [12,13].

Under slit-lamp examination, the presence of Weiss ring aids in the diagnosis of PVD but can be difficult to detect. A previously undescribed sign that can also be visualized in PVD is the anterior hyaloid separation sign (AHSS) which is seen in the anterior vitreous as a "veil of vitreous". The initial separation in PVD occurs superiorly and posteriorly and results in the anterior migration of the cortical vitreous, leaving behind aqueous-filled space posteriorly. This delineation of cortical separation from the retina allows for the visualization of AHSS. Establishing the connection between AHSS and PVD may prove useful for PVD detection. In this study, a retrospective chart review evaluated the presence of AHSS in patients with established PVD.

## Materials and methods

Study design

A retrospective chart review of patients with established PVD (n=214) enrolled at the University of Missouri-Mason Eye Clinic (Columbia, Missouri, United States) between February 1, 2023, and October 1, 2023, was performed.

Ethical considerations

Permission to use patient information in this study was approved by the University of Missouri-Columbia Institutional Review Board (approval number: 403303) prior to accessing patients' electrical medical records. Consent for treatment and open-access publication was obtained or waived by all participants in this study.

Study criteria

Medical charts of all patients were screened for a diagnosis of PVD. PVD cases were defined as patients who had a long-standing history of PVD prior to February 1, 2023, and patients who were diagnosed with PVD within the study time frame. Inclusion criteria were as follows: (1) diagnosis of PVD, (2) presence of Weiss ring under slit-lamp examination, and (3) presence of posterior hyaloid separation confirmed by optical coherence tomography (OCT). PVD patients were stratified as exhibiting AHSS or not having AHSS.

Procedure

All patients presenting to the Mason Eye Clinic followed a routine pupillary dilation with 1% tropicamide and 2.5% phenylephrine prior to ophthalmic evaluation. Patients with a new diagnosis of PVD and a previous history of PVD were evaluated using slit-lamp biomicroscopy, indirect ophthalmoscopy, and OCT.

Assessments

**Figure 1:**
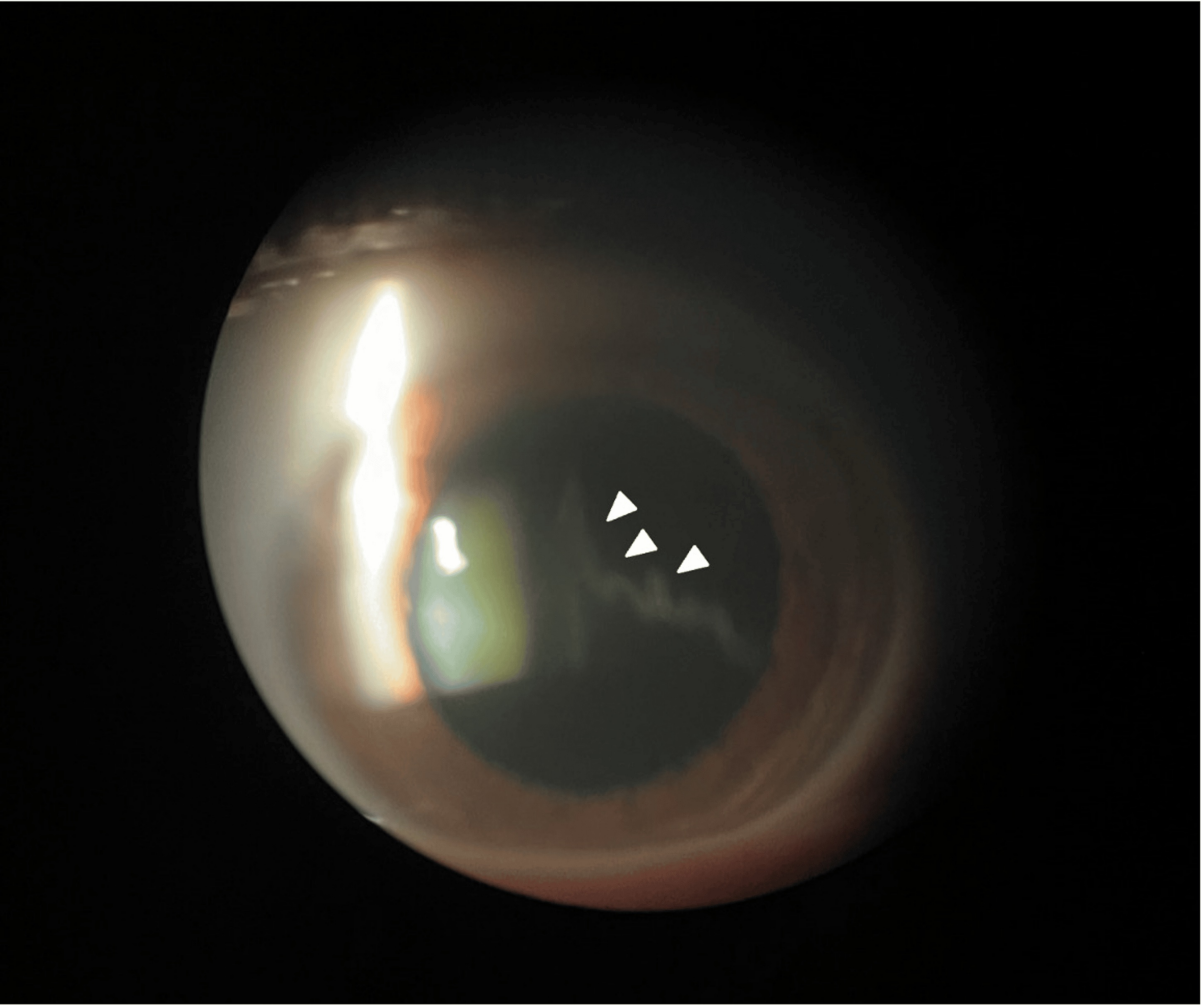
AHSS under slit-lamp biomicroscopy. AHSS can be visualized as a "veil of vitreous" posterior to the lens (white arrow) under slit-lamp biomicroscopy. AHSS: anterior hyaloid separation sign

**Figure 2:**
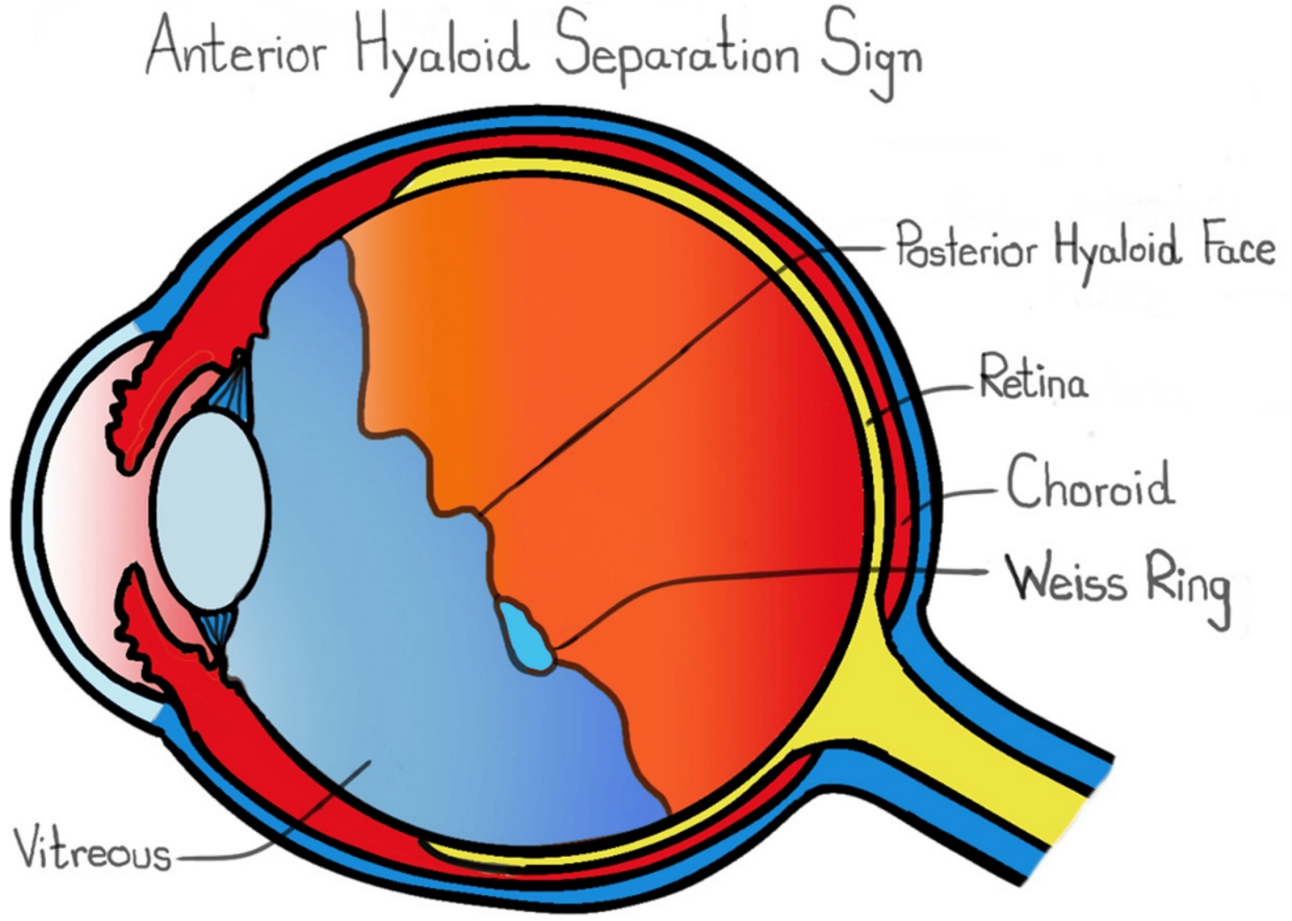
Schematic of AHSS. In PVD, the hyaloid membrane initially separates from the retina superiorly and posteriorly. This results in AHSS and can be visualized through the ophthalmoscope as a "veil of vitreous" of the posterior hyaloid face. AHSS: anterior hyaloid separation sign; PVD: posterior vitreous detachment Image Credits: Peeradol Wattanasirakul

In fully dilated patients, a thin slit beam is angled at 30-45 degrees under slit-lamp biomicroscopy, aiming the focus at the anterior vitreous, which allows for AHSS visualization. AHSS is shown in Figure 1 and the AHSS schematic is depicted in Figure 2. AHSS was first visualized prior to February 1, 2023, and the presence of AHSS within PVD patients was included in the electronic medical record.

Sample size calculation

The sample size was based on the number of patients who met the above-required criteria and presented to the Mason Eye Clinic between February 1, 2023, and October 1, 2023.

Statistical analysis

All data including PVD onset, AHSS status, demographic data (age, sex, race/ethnicity), ocular-related comorbidities, and lens status were transcribed from the electronic medical record (PowerChart) into Microsoft Excel. Ocular-related comorbidity data included diagnoses of proliferative diabetic retinopathy, retinal vessel occlusion, uveitis, sickle cell disease, presence of epiretinal membrane, retinal tear, and RRD. Lens status was categorized as either pseudophakic, phakic, low nuclear sclerotic cataract (nuclear sclerotic cataract below 2+), or high nuclear sclerotic cataract (nuclear sclerotic cataract 2+ and above).

A chi-squared goodness-of-fit test was performed to compare the prevalence of AHSS to the general PVD population. The null hypothesis was defined as no significant difference between the presence and lack of AHSS among PVD patients at 84% prevalence. The alternate hypothesis was defined as a significant difference between the presence and lack of AHSS among PVD patients at 84% prevalence. A p-value of <0.05 was considered significant. Graphs were generated using GraphPad Prism (Version 10, GraphPad Software, La Jolla, California, United States).

## Results

This study examined 214 PVD cases from 147 patients presenting with either bilateral or unilateral PVD with a mean age of 70.3 years (Table 1). The studied population consists of mostly female (72%) and Caucasian patients (93.9%). All demographic profile is detailed in Table 1.

**Table 1:** Demographics of the studied population. PVD: posterior vitreous detachment

Total PVD cases, n	214
PVD patients, n	147
Bilateral PVD	67
Unilateral PVD	80
Mean age in years (SD)	70.3 (9.2)
Sex, n (%)	
Male	60 (28)
Female	154 (72)
Race, n (%)	
Caucasian	201 (93.9)
African-American	6 (2.8)
Asian	2 (1)
Other	5 (2.3)

Lens status and ocular comorbidities of all 214 PVD cases are detailed in Table 2 and Table 3. 

**Table 2:** Lens status of the studied population. *Low NSC: NSC below 2+ **High NSC: NSC 2+ and above NSC: nuclear sclerotic cataract

Lens status	n (%)
Pseudophakic	131 (61.2)
Phakic	3 (1.4)
Low NSC*	35 (16.4)
High NSC**	45 (21)

**Table 3:** Ocular comorbidities of the studied population.

Ocular comorbidities	n (%)
Epiretinal membrane	38 (17.8)
Retinal tear	21 (9.8)
Proliferative diabetic retinopathy	11 (5.1)
Retinal vein occlusion	10 (4.7)
Uveitis	10 (4.7)
Rhegmatogenous retinal detachment	9 (4.2)
Sickle cell disease	3 (1)

**Figure 3:**
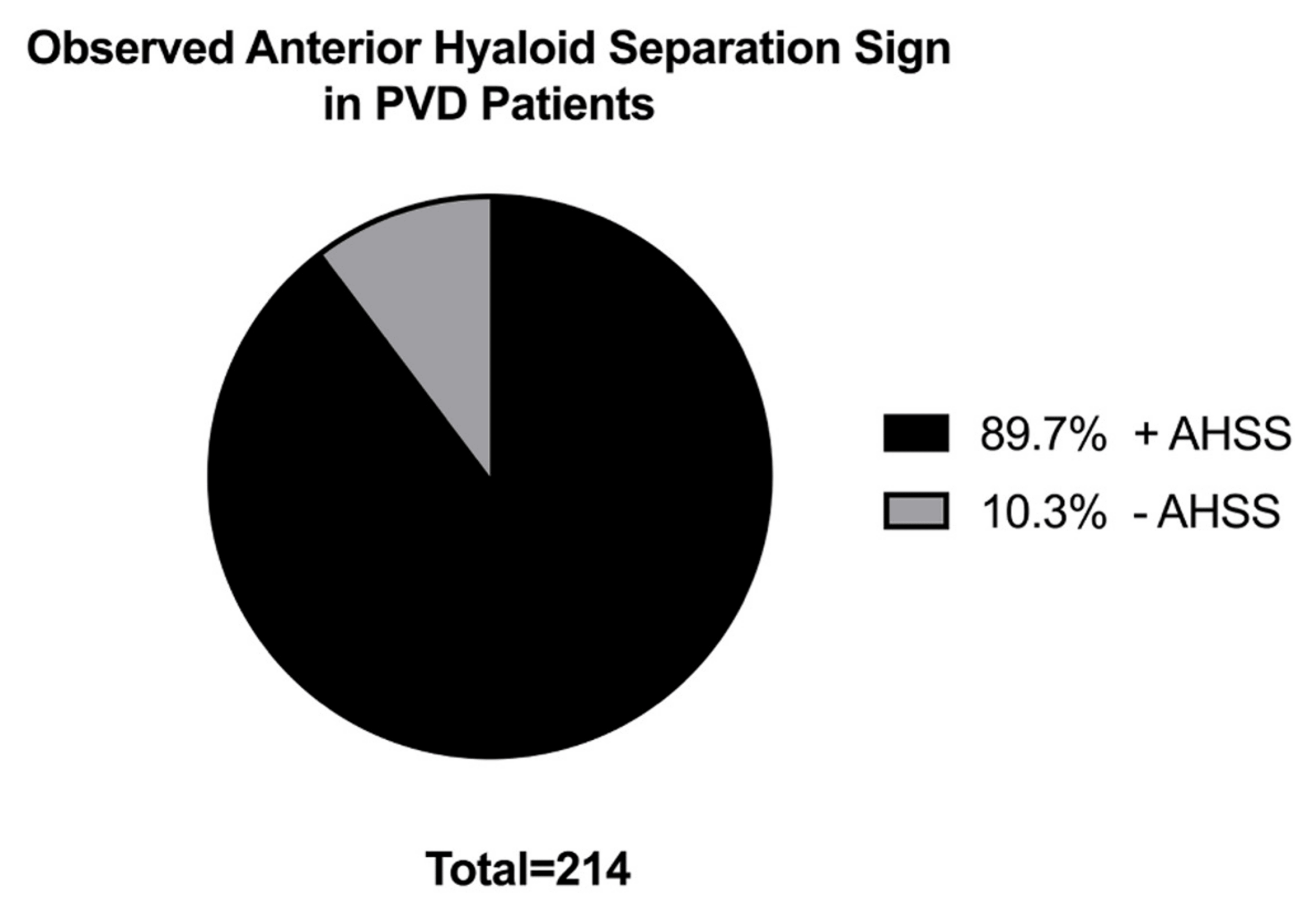
Presence of AHSS among PVD patients. Within the PVD population, 89.7% (n=192) exhibit AHSS and 10.3% (n=22) did not exhibit AHSS. AHSS: anterior hyaloid separation sign; PVD: posterior vitreous detachment

Among all PVD cases, 89.7% (n=192) presented with AHSS and 10.3% (n=22) did not present with AHSS (Figure 3).

**Figure 4:**
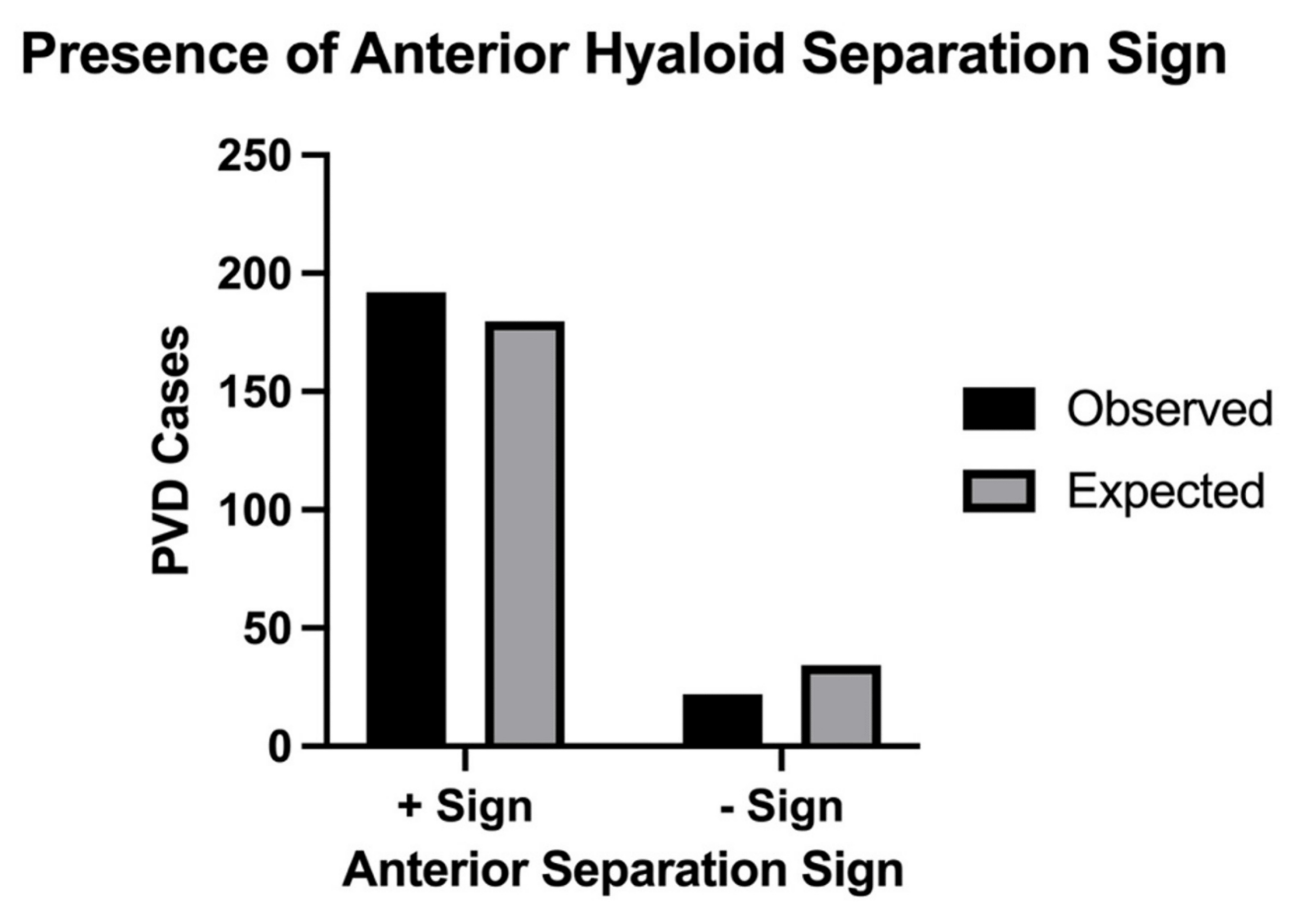
Observed versus expected presence of AHSS in PVD patients. The chi-squared goodness-of-fit test compared the observed versus expected prevalence of AHSS in PVD patients at 84% prevalence. Analysis resulted in p=0.02. Significant difference between the presence and absence of AHSS among PVD patients is at 84% prevalence. AHSS: anterior hyaloid separation sign; PVD: posterior vitreous detachment

The chi-squared goodness-of-fit test resulted in p=0.02. Analysis reveals a significant difference between the presence and absence of AHSS among PVD patients at 84% prevalence. A representation of observed versus expected AHSS in PVD patients at 84% prevalence is depicted in Figure 4. Of those exhibiting AHSS, 56.2% had a new diagnosis of PVD (n=108), and 43.8% had long-standing PVD (n=84). In long-standing PVD cases, the median time between initial PVD diagnosis and recorded AHSS detection is 350 days. A further look at PVD patients without AHSS did not show any significant variable to suggest an explanation for the absence of the sign.

## Discussion

In current practices, PVD diagnosis is aided by image-based methods (B-scan ultrasonography and OCT) [14] and the visualization of Weiss ring under slit-lamp microscopy [15] or indirect ophthalmoscopy [16,17]. Previously, studies of similar size reported on the prevalence of Weiss ring in patients with PVD. Akiba et al. reported a 93.3% prevalence of Weiss ring among 223 PVD cases [16], while Kakehashi et al. reported 51% complete Weiss ring and 36% incomplete Weiss ring among 200 PVD cases [17]. This study aims to explore a connection between the presence of AHSS among PVD cases, and the data indicates that 89.7% of the PVD population (n=214) exhibit AHSS.

A chi-squared goodness-of-fit analysis was performed to compare this study's PVD population to the general PVD population. The analysis reveals a significance of 84% prevalence of AHSS among PVD cases (p=0.02). The discrepancy between observed AHSS in this study (89.7%) and the predicted AHSS prevalence in the general PVD population (84%) is due to the low power of this study (n=214). In our population, among PVD patients presenting with AHSS, 43.8% (n=84) had a previous diagnosis of PVD. The median time between PVD diagnosis and AHSS detection is 350 days. This suggests that AHSS can help confirm PVD in both acute and long-standing cases.

Although not seen in all PVD cases, Weiss ring is still regarded as an important index for PVD diagnosis. The data in this study suggests a similar claim, that is, the presence of AHSS holds a significant value as an indicator of PVD. AHSS can be used in conjunction with clinical presentations and signs of PVD to aid in the diagnosis and confirmation of PVD.

The detection of Weiss ring requires an unobstructed view of the posterior segment using indirect slit-lamp biomicroscopy. Visualization of the posterior segment can prove challenging if vitreous hemorrhage or other media opacity such as high-grade cataract is present. The difficulty in mastering this process makes Weiss ring detection highly user dependent. Unlike the detection of Weiss ring, AHSS can be identified by practitioners of any expertise level as it can be seen without the use of a lens.

Further studies should investigate the common factors among PVD patients without AHSS. In this study, only 10.3% (n=22) of PVD patients did not present with AHSS. These patients presented with varying types of ocular-related comorbidities. We suspect that ocular conditions like proliferative diabetic retinopathy, central retinal vein occlusion, proliferative sickle cell retinopathy, and uveitis may be related to the lack of AHSS. These conditions are characterized by increased inflammatory activities through the release of cytokines and chemokines which alter the retinal microenvironment. We suspect that this proinflammatory state may increase adhesions between the posterior hyaloid face and the internal limiting membrane, restricting the anterior migration of the vitreous and preventing the visualization of AHSS. Other conditions like epiretinal membranes, retinal tears, and RRD are also reflective of stronger vitreoretinal adhesions that can potentially limit the extent of anterior separation of the vitreous. As noted in Table 3, these ocular comorbidities were identified among PVD patients, but the sample size of this study is too small for adequate power to infer any relationship.

Another possible factor in the lack of AHSS among PVD patients is the physical limitation of viewing AHSS. Detecting AHSS is dependent on the clear visualization of the anterior segment which is difficult if the pupil is not fully dilated. While patients presenting to the Mason Eye Clinic were dilated with 1% tropicamide and 2.5% phenylephrine, accurate data on pupil size could not be gathered from the medical record. The challenge in viewing the anterior segment could also be due to the cataract severity of each PVD case. As noted in Table 3, an attempt to categorize the lens status of all patients was made, but a complete cataract evaluation was not present in the electronic medical record. The retrospective nature of the study limits the type of information that can be gathered which presents a challenge in establishing common factors among PVD patients without AHSS. Further prospective studies should be sought to answer these questions.

## Conclusions

PVD is an age-related ocular condition characterized by the separation of the vitreous humor from the retinal surface and presents with a range of clinical manifestations that warrant careful consideration. Current practice utilizes the detection of Weiss ring to aid in PVD diagnosis; however, it can be difficult to detect. AHSS is a previously undescribed sign also seen among patients with PVD. The data of this study indicates a large prevalence of AHSS (89.7%) among PVD patients suggesting that AHSS can help aid in the diagnosis of PVD. Future prospective studies should investigate if common factors exist among the PVD population who do not present with AHSS.
